# Effects of Tea Seed Oil Extracted by Different Refining Temperatures on the Intestinal Microbiota of High-Fat-Diet-Induced Obese Mice

**DOI:** 10.3390/foods13152352

**Published:** 2024-07-26

**Authors:** Lin Chen, Qihong Jiang, Hongling Lu, Chenkai Jiang, Wenjun Hu, Hanxiao Liu, Xingwei Xiang, Chin Ping Tan, Tianhuan Zhou, Guoxin Shen

**Affiliations:** 1 Institute of Sericultural and Tea, Zhejiang Academy of Agricultural Sciences, Hangzhou 310021, China; chenlinsdau@163.com (L.C.); jiangqihong2020@163.com (Q.J.); luhongling@zaas.ac.cn (H.L.); greenbreezekai@126.com (C.J.); guyuexingshi@163.com (W.H.); 2 Zhejiang Feida Environmental Science & Technology Co., Ltd., Shaoxing 311800, China; gutounan@163.com; 3 College of Food Science and Technology, Zhejiang University of Technology, Hangzhou 310014, China; xxw11086@zjut.edu.cn; 4 Department of Food Technology, Faculty of Food Science and Technology, University Putra Malaysia, Serdang 43400, Malaysia; tancp@upm.edu.my; 5 Zhejiang Forest Resources Monitoring Center, Hangzhou 310020, China

**Keywords:** tea seed oil, oil refining process, high-fat diet, intestinal bacteria

## Abstract

Obesity has become one of the most serious chronic diseases threatening human health. Its onset and progression are closely related to the intestinal microbiota, as disruption of the intestinal flora promotes the production of endotoxins and induces an inflammatory response. This study aimed to investigate the variations in the physicochemical properties of various refined tea seed oils and their impact on intestinal microbiota disorders induced by a high-fat diet (HFD) through dietary intervention. In the present study, C57BL/6J mice on a HFD were randomly divided into three groups: HFD, T-TSO, and N-TSO. T-TSO and N-TSO mice were given traditionally refined and optimized tea seed oil for 12 weeks. The data revealed that tea seed oil obtained through degumming at 70 °C, deacidification at 50 °C, decolorization at 90 °C, and deodorization at 180 °C (at 0.06 MPa for 1 h) effectively removed impurities while minimizing the loss of active ingredients. Additionally, the optimized tea seed oil mitigated fat accumulation and inflammatory responses resulting from HFD, and reduced liver tissue damage in comparison to traditional refining methods. More importantly, N-TSO can serve as a dietary supplement to enhance the diversity and abundance of intestinal microbiota, increasing the presence of beneficial bacteria (*norank_f__Muribaculaceae*, *Lactobacillus*, and *Bacteroides*) while reducing pathogenic bacteria (*Alistipes* and *Mucispirillum*). Therefore, in HFD-induced obese C57BL/6J mice, N-TSO can better ameliorate obesity compared with a T-TSO diet, which is promising in alleviating HFD-induced intestinal microbiota disorders.

## 1. Introduction

Obesity, a chronic condition characterized by excessive fat storage or abnormal fat distribution [1], has become increasingly prevalent in recent years due to changes in dietary habits and improved living standards. According to the latest data from the World Obesity Federation project that by 2035, over 4 billion individuals may be overweight or obese, with obesity prevalence expected to surge from 14% in 2020 to 24%, affecting nearly 2 billion people [2]. The mechanism of obesity pathogenesis is the excess of energy intake over energy expenditure. Obesity is the result of the interaction of multiple causes including genetic factors, environmental factors, abnormal endocrine regulation, inflammation, and gut flora. In the context of HFD, damaged intestinal barrier, activated inflammatory signaling pathways, mitochondrial dysfunction, and pathogenic antibiotic resistance cause local and systemic inflammation [3]. More seriously, maternal HFD even significantly increases lipid content and blocks the AMPK/SIRT1/PGC-1α signaling pathway in the offspring, leading to an increased risk of obesity [4]. Importantly, obesity can lead to various health complications, including hypertension, hyperlipidemia, hyperglycemia, inflammation, disruptions in gut homeostasis, and an elevated risk of cancer [5,6,7]. Currently, established approaches to combat obesity includes pharmacological interventions, dietary adjustments, and calorie restriction to reduce fat accumulation. Common long-term weight loss medications include orlistat, naltrexone-bupropion, phentermine-topiramate, or liraglutide [8], but their prolonged use can result in varying degrees of side effects [9]. Lifestyle modifications, such as energy limitation and alterations in macronutrients and dietary intake, stand as effective strategies for improving obesity [10].

An increasing body of research has highlighted the potential of vegetable oils rich in medium-chain fatty acids (MCFAs) to enhance lipid metabolism by lowering total cholesterol (TC) and low-density lipoprotein (LDL) levels while restraining body weight (BW) and body mass index (BMI) gain [11]. Tea seeds, which are the seeds of the tea tree [*Camellia sinensis* (L) O. Ktze], have garnered attention since 2009 when tea seed oil officially gained recognition as a novel food resource. Tea seed oil (TSO) represents a woody edible oil extracted from mature tea seeds and its fatty acid composition closely resembles that of olive oil. Notably, its monounsaturated fatty acid (MUFA) content surpasses that of olive oil, aligning with international nutritional standards such as the Omega Diet [12]. Tea seeds contain 20–30%, primarily consisting of oleic acid, linoleic acid, palmitic acid, and stearic acid, which collectively constitute over 90% of the total fatty acids [13]. Furthermore, tea seed oil contains a variety of bioactive components, including polyphenols, vitamin E, squalene, and flavonoids [14]. Recent studies have unveiled tea seed oil’s potential to improve cognitive function, lower blood pressure and lipid levels, enhance gastrointestinal absorption, and prevent cardiovascular disease [15]. Therefore, tea seed oil holds promise as a high-quality edible oil for promoting health.

Current research on tea seed oil primarily focuses on oil extraction, processing, and the determination of its bioactive constituents. The primary methods used for tea seed oil extraction in production include hot pressing, cold pressing, and solvent extraction. However, solvent extraction has gradually fallen out favor due to environment concerns. Despite the lower oil yield achieved through pressing, this method is increasingly prevalent in the edible oil production because it inflicts minimal damage on oil quality [16]. Crude tea seed oil obtained through pressing has an unpalatable bitterness and contains phospholipids necessitating the refining process to make it suitable for human consumption. Conventional refining, which typically employs high temperatures (degumming at 75–80 °C, neutralization at 80–90 °C, bleaching at 110–130 °C under a vacuum of 0.4 kPa, and deodorization at 240–260 °C), serves to eliminate impurities but also eliminates most of the bioactive compounds, thereby diminishing its value [17]. Consequently, we optimized the refining temperature of tea seed oil and employed a high-fat diet to induce obesity in mice, allowing us to investigate the influence of various refining methods on its lipid-lowering properties. This study fills the gap in the process of refining tea seed oil to retain active substances and offers a theoretical foundation and a reference point for the potential use of tea seed oil in ameliorating obesity.

## 2. Materials and Methods

### 2.1. Materials

Tea seeds (*Camellia Sinensis* O.Ktze) were sourced from Xi Shuang Banna (Yunnan, China). They were processed using screw-type seed presses by the Zhejiang Academy of Agricultural Sciences and filtered to obtain cold-pressed tea seed crude oil (Hangzhou, China). We utilized 37 chromatographically pure fatty acid standards, acquired from Sigma Corporation, USA. A tocopherol standard mixture (95% purity, containing α, β, γ, and δ) was obtained from Roche (Wakaville, CA, USA). All experimental animal diets were purchased from Suzhou Shuangsi Experimental Animal Feed Technology Co. (Suzhou, China). Analytical grade chemical reagents were employed for this study.

### 2.2. Oil Refining Procedures

In the laboratory, 12 oil samples (1 kg each) underwent consecutive degumming, deacidification, decolorization, and deodorization refining steps. Each refining step was carried out at five temperature treatments, with each step repeated three times. In each refining stage, 100 g of oil was taken for quality analysis, while the remaining portion proceeded to the next step. The optimal refining process was determined based on the experiment results, with detailed methods provided in the Supplementary Material of this manuscript. For the factory-based refining experiments, 50 kg crude oil samples underwent refining: the first sample was refined using the traditional refining method (degumming at 85 °C, deacidification at 65 °C, decolorization at 120 °C, and deodorization at 260 °C under a vacuum of 0.14 MPa for 3 h); the second sample was refined using the optimized refining method (degumming at 70 °C, deacidification at 50 °C, decolorization at 90 °C, and deodorization at 180 °C under a vacuum of 0.06 MPa for 1 h). The tea seed oil produced via the traditional refining method was labeled as T-TSO, and the optimized refined tea seed oil was named N-TSO.

### 2.3. Fatty Acid Determination

The determination of fatty acid followed the national standard GB 5009.168-2016 of the People’s Republic of China [18]. To produce fatty acid methyl esters (FAME), 0.1 g of the sample was dissolved in 4 mL of an iso-octane solution, followed by the addition of 340 μL of a 2 mol/L potassium hydroxide methanol solution. The mixture was vigorously shaken for 1 min and allowed to stand for 30 min until it became clear. Accelerated separation was achieved by adding 5 mL of saturated saline, and the supernatant was collected and filtered through a 0.22 μm membrane for subsequent analysis. The fatty acid composition of the oil was determined using a gas chromatography (GC) analyzer (Agilent 7890A, Santa Clara, CA, USA) equipped with a flame ionization detector (FID) and an HP-5MS capillary column (30 m, 250 μm, 0.25 μm). A 1 μL oil sample was injected into the column through an autosampler with a 1:60 split ratio, and hydrogen was used as the carrier gas. The column temperature was maintained at 60 °C for 1 min after injection, then gradually increased to 280 °C at a rate of 5 °C/min and held for 2 min. Fatty acid peaks were identified by comparing their relative retention times with those of the FAME standard mixture.

### 2.4. Determination of Active Ingredients

#### 2.4.1. Total Polyphenols

Polyphenols from tea seed oil were extracted following the method outlined by Wang et al. [19]. In brief, 2.5 g of the oil sample was weighed into a centrifuge tube, and 6 mL of hexane and 6 mL of methanol–water (60:40, *v*/*v*) were added. The mixture was vortexed for 3 min, then centrifuged at 4 °C for 10 min at 3500 g. The methanol solution was collected through multiple centrifugation separations and allowed to evaporate to dryness at 35 °C. Finally, the residue was dissolved in 250 μL of methanol–water (50:50, *v*/*v*) and filtered through a 0.2 μm organic membrane for further analysis. The determination of tea polyphenol content followed the national standard LS/T 6119-2017 of the People’s Republic of China [20].

#### 2.4.2. Tocopherols and Squalene

The determination of tocopherol and squalene followed the method reported by Li et al. [21]. A 200 mg oil sample was accurately weighed and added to 3 mL of a 0.5 mol/L NaOH–ethanol solution. The mixture was vortexed for 30 s and saponified at 70 °C for 40 min with shaking every 5 min. After reaching room temperature, 2 mL of water and 3 mL of hexane were added, vortexed for 30 s, and the supernatant was collected. This extraction was repeated three times, and the supernatant was combined and dried using nitrogen. The resulting supernatant was re-dissolved in 1 mL of a methanol: isopropanol (*v*:*v* = 1:1) solution, filtered through a 0.22 µm membrane, and analyzed by HPLC on a Waters SunFire C18 reversed-phase chromatographic column (4.6 mm × 250 mm, 5 μm). The liquid-phase conditions included a column temperature of 30 °C, a mobile phase of methanol: isopropanol (*v*:*v* = 9:1) at a flow rate of 1 mL/min, and an injection volume of 10 μL, with dual-wavelength detection at 205 nm and 296 nm.

#### 2.4.3. Sterols and Carotenoids

The sterol content in tea seed oil was determined following previous reports [17]. A sample weighing 0.1 g was accurately weighed, and the concentration was adjusted to 25 mL with n-hexane. It was then placed in a cuvette, and the absorbance was determined at 446 nm using a UV spectrophotometer with a blank solvent as a reference. The carotenoids were calculated using the formula: C=383E/IG, where *E* represents the absorbance of the sample, *I* is the width of the cuvette in cm, *G* stands for the content of tea seed oil in the sample in g/100 mL, and *C* represents the carotene content in mg/kg.

### 2.5. Physical and Chemical Property Analysis

The Chinese National Standard for Tea Seed Oil (CNS-TSO) [22] was used to analyze the chemical and physical properties of tea seed oil. The refractive index was determined using an Abbe refractometer (Shanghai Jiehu Instrumentation Co., Ltd., Abbe 60/LR, Shanghai, China). Meanwhile, a 200 mg oil sample was treated with 25 mL of Wijs solution in a conical flask and left to stand for 2 h away from light. The iodine concentration was determined by titration with sodium thiosulfate after adding 20 mL of potassium iodide solution and 150 mL of water. Phospholipid content was determined using the molybdenum blue colorimetric method. The acid value was determined following a previously established laboratory method. For the peroxide value, moisture, and volatiles, we employed standard methods with some modifications from previous research [17]. 

### 2.6. Animal Experimental Design

We procured twenty-four 4-week-old male SPF-grade C57BL/6J mice (17 ± 1 g) from Hangzhou Zhiyuan Experimental Animal Science and Technology Co Ltd. (Hangzhou, China). All mice were accommodated in a non-pathogenic animal room maintained at 22~24 °C with 55% relative humidity and a 12-h dark/light cycle for one week as an acclimatization period. Subsequently, the mice were randomly assigned to four groups (n = 6): the control group (NC, normal chow + distilled water), the model group (HFD, high-fat diet + distilled water), the traditional refined tea seed oil group (T-TSO, high-fat diet + 225 mg/kg BW/day T-TSO), and the optimized refined tea seed oil group (N-TSO, high-fat diet + 225 mg/kg BW/day N-TSO). All mice received the intervention through oral gavage during the experimental period, and their weights were regularly recorded weekly. According to Aldamarany et al. [23], the daily oil consumption of 225 mg/kg/d in mice is equivalent to 18.23 mg/kg/day in adults (body weight 60 kg) and 27 mg/kg/day in children (body weight 20 kg), which was significantly lower than the daily dose recommended by the Chinese Nutrition Society (25–30 g/day). This animal experiment was reviewed and approved by the Institutional Animal Care and Use Committee of the Zhejiang Academy of Agricultural Sciences (2022ZAASLA86).

After 12 weeks, the mice were anaesthetized via intraperitoneal injection of isoflurane, and blood samples were collected by cardioplegia. Isoflurane offers many advantages in murine anesthesia, including easy titration of the dose and depth of anesthesia and rapid induction of and recovery from anesthesia [24]. More importantly, isoflurane does not cause significant differences in biochemical indices [25]. The blood samples were allowed to stand at 2 °C for 4 h, then centrifuged at 3000 rpm for 10 min, and the serum was collected and stored at −80 °C. Furthermore, liver and adipose tissue (epididymis, perirenal and subcutaneous fat) were collected and weighed. A portion of the liver and epididymal fat was preserved in formalin for subsequent hematoxylin and eosin (H&E) staining. Additionally, cecum contents from mice were collected and stored at −80 °C for gut microbiology analysis.

### 2.7. Measurement of Serum Biochemical Indicators

Serum levels of triglyceride (TG), total cholesterol (TC), low-density lipoprotein cholesterol (LDL-C), high-density lipoprotein cholesterol (HDL-C), diamine oxidase (DAO), tumor necrosis factor-alpha (TNF-α), interleukin-1β (IL-1β), and lipopolysaccharide (LPS) were determined as per the kit instructions. TG, TC, LDL-C, and HDL-C kits were obtained from Nanjing Jiancheng Bioengineering Co. Ltd. (Nanjing, China); TNF-α, IL-1β, LPS, and DAO kits were purchased from Wuhan Boster Biological Technology., Ltd. (Wuhan, China).

### 2.8. H&E Staining of Liver Tissue and Epididymal Fat

Liver and epididymal fat samples from each group of mice were fixed in 4% formaldehyde, embedded in paraffin, and subjected to H&E staining [26]. The changes in liver tissue were examined under a light microscope.

### 2.9. RT-PCR Analysis

Total RNA was extracted from liver tissue using Trizol reagent. Subsequently, RNA was reverse transcribed into cDNA using a cDNA synthesis kit. RT-PCR analysis was conducted on a StepOnePlus RT-PCR detection system (Zhejiang Scientific Instruments and Materials I/E Co., Ltd., Zhejiang, China) with SYBR Green PCR Master Mix. β-actin served as a control housekeeping gene and was quantified using the 2^−ΔΔCt^ method. Trizol reagent, total RNA extraction kit, and cDNA synthesis kit were purchased from Tiangen Biochemical Technology (Beijing) Co., Ltd. (Beijing, China); SYBR Green PCR Master Mix was purchased from Yisheng Biotechnology Shanghai Co., Ltd. (Shanghai, China). RT-PCR primer sequences are provided in the Supplementary Material.

### 2.10. 16 S rRNA Sequencing for Gut Microbiota

The composition of the mouse gut microbial was assessed following established methods [27]. Total genomic DNA of the intestinal microbiota was extracted from the cecal contents of mice using the MOBIO PowerSoil^®^ DNA Isolation kit (MOBIO, Carlsbad, CA, USA). The integrity and quality of the DNA were assessed using gel electrophoresis (1% agarose). Primer sequences (F: 5′-ACTCCTACGGGAGGCAGCAG-3′ and R: 5′-GGACTACHVGGGTWTCTAAI-3′) were used to amplify the V3–V4 hypervariable region of the bacterial 16S rRNA gene. Sequencing was performed on the Illumina Miseq PE300 platform (Shanghai Majorbio Bio-pharm Technology Co., Ltd., Shanghai, China). Raw sequences were processed using fastp QC, with sequences being trimmed and optimized using FLASH software (Version 34.0.0.317). Then, sequence reads with over 97% similarity were clustered into multiple chimera-free operational taxonomic units (OTUs) utilizing UPARSE software (Version 3.1.0). α-diversity analysis, principal coordinate analysis (PCoA), β-diversity analysis, and microbial taxon distribution analysis were performed using Mothur software (Version V1.45.3), Qiime software (Version 2024.2), and R software (Version R-4.3.2).

### 2.11. Statistical Analysis

Experimental data were expressed as mean ± standard deviation (SD). One-way analysis of variance (ANOVA) was used to evaluate differences between groups, followed by Duncan’s test or Tukey’s post hoc test. All statistical analyses were performed using GraphPad Prism 7.0 software (La Jolla, CA, USA) and SPSS 19.0 (SPSS Inc., Chicago, IL, USA).

## 3. Results and Discussion

### 3.1. Effect of Different Refining on Oil Physicochemical Properties

Tea seeds, with their oil content of 30–32%, are primarily composed of monounsaturated fatty acid (MUFA) oleic acid and polyunsaturated fatty acid (PUFA) linoleic acid [28], making them a valuable source of high-quality edible oil. Oleic acid, known for its positive impact on reducing LDL-C and TC levels, is believed to aid in addressing obesity [29]. However, crude tea seed oil extracted through pressing contains substantial amounts of bitter compounds and phospholipids, necessitating refining to render make it safe for human consumption as an edible oil. It is crucial to not only effectively remove phospholipids, colors, odors, and other impurities during the refining process, but also to prevent the thermal decomposition of oleic acid and other bioactive compounds. As presented in Table 1, we have assessed the key characteristics of the resulting oils produced under varying refining conditions, including fatty acid composition, chemical quality indicators, following the commercial quality standards of CNS-TSO. The data revealed that tea seed oil produced under optimal conditions exhibited a reduced acid value (AV) to 0.21 mg KOH/g, peroxide value (POV) to 0.87 mmol kg^−1^, and a phospholipid content of 0.017%. Additionally, the moisture, volatiles, and insoluble impurities content after refining met CNS-TSO standards.

Furthermore, there was no significant difference (*p* > 0.05) in the fatty acid composition and content of tea seed oil prepared through different refining processes, with unsaturated fat constituting over 80% (Figure S1), aligning with the results obtained via supercritical carbon dioxide extraction [16]. Significantly, indigestible or harmful fatty acids such as erucic acid and behenic acid were undetectable in TSO, thereby eliminating the potential health risks associated with heart damage [30] (Table 1). Vegetable oils, which are rich in a variety of unsaturated fatty acids, have been reported to reduce cholesterol and triglyceride levels, improve dyslipidemia, and show great potential in mitigating the development of atherosclerosis [31]. In contrast to crude oil, traditional tea seed oil refining effectively removed bitterness and impurities, but the high-temperature treatment resulted in significant loss of bioactive compounds [32]. Among these, 79.48% of total polyphenols, 75.24% of total tocopherols, 74.79% of sterols, 73.14% of squalene, and 82.26% of carotenoids were lost. In comparison, oil refined using the optimized method resulted in the loss of only 18.70% for total polyphenols, 15.12% for total tocopherols, 10.41% for sterols, 16.01% for squalene, and 26.97% for β-carotene (Table 2).

### 3.2. Effect of Different Refined TSO on BW and Fat Content in Mice

**Figure 1:**
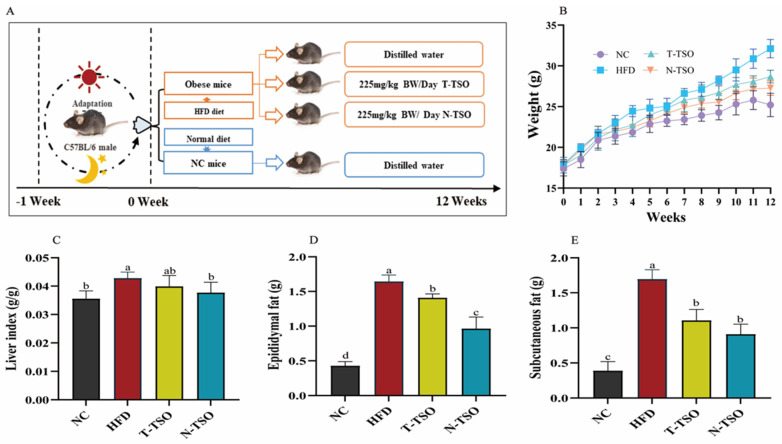
Effect of different refined tea seed oils on body weight of obese mice induced by high-fat diet. (**A**) Classification of five diet treatment groups for a 12-week feeding cycle, (**B**) body weight, (**C**) liver index, (**D**) epididymal fat, (**E**) subcutaneous fat. Data are presented as the mean ± SD (n = 6). Different letters represent a significant difference among multiple groups (*p* < 0.05). NC: control group, HFD: model group, T-TSO: traditional refined tea seed oil group, N-TSO: optimized refined tea seed oil group.

To investigate the inhibitory effects of various refined tea seed oils on obesity, we administered T-TSO and N-TSO as dietary supplements to mice fed a high-fat diet. Excessive energy consumption leads to the overfilling and hypertrophy of fat cells, causing metabolic disturbances and health problems [33]. Compared to their initial body weight, the NC, HFD, T-TSO, and N-TSO groups exhibited a weight gain of 46.45%, 80.00%, 60.99%, and 53.04%, respectively. Importantly, mice in the gavage T-TSO groups experienced an 11.06% reduction in body weight, and those in the N-TSO group experienced a 15.45% reduction compared to the HFD group (Figure 1B). Moreover, Figure 1C–E indicated that tea seed oil significantly (*p* < 0.05) reduced fat accumulation and inhibited weight gain, consistent with previous research findings [34]. The fat reduction was more pronounced with N-TSO, which may be attributed to the higher content of active ingredients in tea seed oil.

### 3.3. Effect of Different Refined TSO on Dyslipidemia in Obese Mice

**Figure 2:**
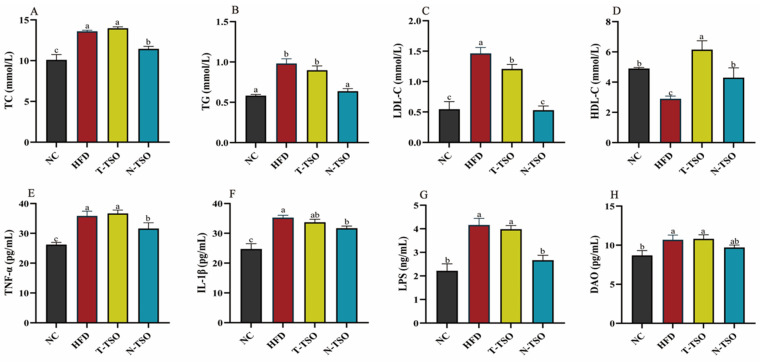
Effect of different refined tea seed oils on serum biochemical indicators of obese mice induced by high-fat diet. (**A**) TC, (**B**) TG, (**C**) LDL-C, (**D**) HDL-C, (**E**) TNF-α, (**F**) IL-β, (**G**) LPS, and (**H**) DAO. Data are presented as the mean ± SD (n = 6). Different letters represent a significant difference among multiple groups (*p* < 0.05). NC: control group, HFD: model group, T-TSO: traditional refined tea seed oil group, N-TSO: optimized refined tea seed oil group.

Hyperlipidemia is a prevalent feature in obese individuals, characterized by elevated TC, TG, and LDL-C levels, and reduced HDL-C. As shown in Figure 2A–C, serum levels of TC, TG, and LDL-C increased significantly (*p* < 0.05), while HDL-C was decreased in mice on a chronic high-fat diet. Dyslipidemia is primarily attributed to the saturation of adipose tissue storage capacity and the rise of triglyceride-rich chylomicrons in bloodstream [33]. TSO, rich in oleic acid, which is beneficial for normalizing lipid metabolism and lowering the risk of cardiovascular diseases [35], effectively reversed this phenomenon in both HFD group. Notably, N-TSO was more effective, reducing TC by 15.88%, TG by 35.07%, and LDL-C by 63.86% compared to the HFD group. This improvement could be attributed to the higher content of polyphenols, sterols, and squalene in N-TSO. Research has suggested that phytosterols and polyphenols can reduce LDL-C levels by inhibiting intestinal absorption, positively impacting fat accumulation and obesity [32]. In the long run, both tea seed oils attenuated the development of obesity and atherosclerosis, with a more pronounced role for N-TSO in ameliorating obesity.

### 3.4. Effect of Different Refined TSO on Inflammation in Obese Mice

Obesity-induced inflammation is a key characteristic of adipose tissue dysfunction. An abnormal balance of various adipokines and metabolites in adipose tissue triggers the activation of monocytes, leading to the secretion of inflammation-associated adipokines [36]. In this study, serum TNF-α and IL-1β levels were significantly (*p* < 0.05) higher in the HFD group compared to the NC group, increasing by 36.66% and 42.56%. However, intervention with optimally refined TSO significantly (*p* < 0.05) suppressed the obesity-induced elevation of inflammatory cytokines TNF-α and IL-1β (Figure 2E,F), decreasing by 11.77% and 10.05%, respectively. Obesity is closely linked to elevated blood levels of LPS, increased intestinal permeability and intestinal disorders [37]. Consequently, we measured serum levels of LPS and DAO in mice to evaluate intestinal damage induced by HFD-induced obesity. The results revealed that N-TSO treatment significantly (*p* < 0.05) reduced serum LPS and DAO levels in mice compared to the HFD group (Figure 2G,H) and reduced them by 35.95% and 9.27%, respectively.

### 3.5. Observation of Adipose Tissue Morphology

**Figure 3:**
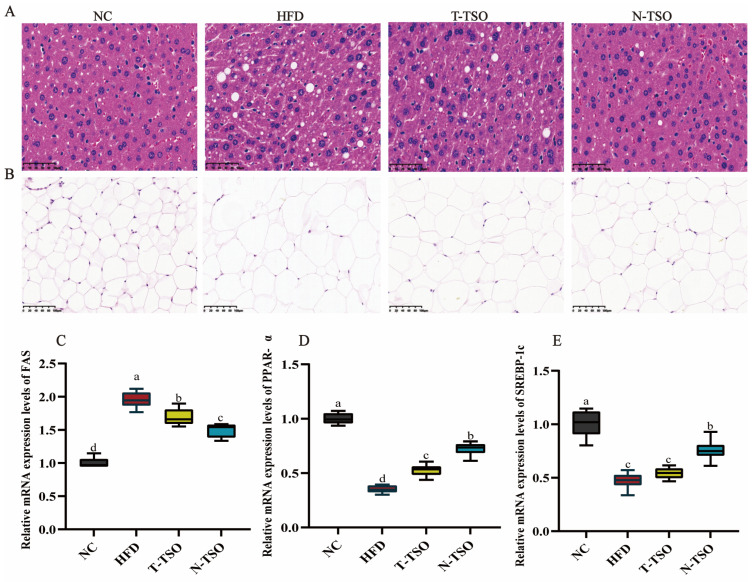
Pathological observations and expression of lipid metabolism-related genes in different groups of obese mice. (**A**) H&E staining of liver tissue, (**B**) H&E staining of epididymal fat, (**C**) FAS, (**D**) PPAR-α, and (**E**) SREBP-c. Data are presented as the mean ± SD (n =3). Different letters represent a significant difference among multiple groups (*p* < 0.05). Scale bar = 50 and 100 μm.

As a vital organ of body’s lipid metabolism, the liver plays a crucial role in regulating lipid homeostasis [27]. H&E staining showed that the HFD diet caused disorganization and an increase in the size of hepatocytes, along with the production numerous cytosolic vesicles. Simultaneously, epididymal fat cells enlarged and lost their regularity. After N-TSO intervention, hyperlipidemic mice displayed a significant (*p* < 0.05) reduction in liver injury, such as a decrease in cytosolic vesicles and a more regular cell arrangement. Furthermore, adipocytes exhibited structural normalcy and regular arrangement, consistent with the results of serum indexes and adipose tissue weight (Figure 3A,B). Notably, N-TSO was more effective in preventing lipid accumulation and ameliorating obesity compared to T-TSO.

### 3.6. Effect of Different Refined TSO on Lipid Metabolism Genes

To further investigate the regulatory mechanism of TSO on lipid metabolism, we assessed the mRNA expression levels of fatty acid synthase (FAS), peroxisome proliferator-activated receptor-α (PPAR-α), and sterol regulatory element binding protein-c (SREBP-c) using real-time quantitative PCR. SREBP-1c is a transcription factor that governs cholesterol biosynthesis and the expression of lipogenic gene (ACC and FAS) [38]. When there is disordered lipid metabolism in vivo, SREBP-1c binds to the promoter region of its target genes to promote the transcription of lipogenic enzymes, consequently stimulating lipid synthesis. Furthermore, PPAR-α increases cellular fatty acid uptake, esterification, and transport, while reducing free fatty acid levels [39]. Compared with the NC group, the mRNA expression levels of FAS were significantly elevated in the HFD group of mice, and the mRNA expression levels of PPAR-α and SREBP-c were significantly (*p* < 0.05) decreased. Following T-TSO intervention, FAS levels decreased significantly, and SREBP-c increased slightly. However, all these trends were markedly reversed after N-TSO intervention (Figure 3C–E), aligning with the previous serum indexes and H&E staining results.

### 3.7. Effect of Different Refined TSO on Intestinal Microbiota of Obese Mice

**Figure 4:**
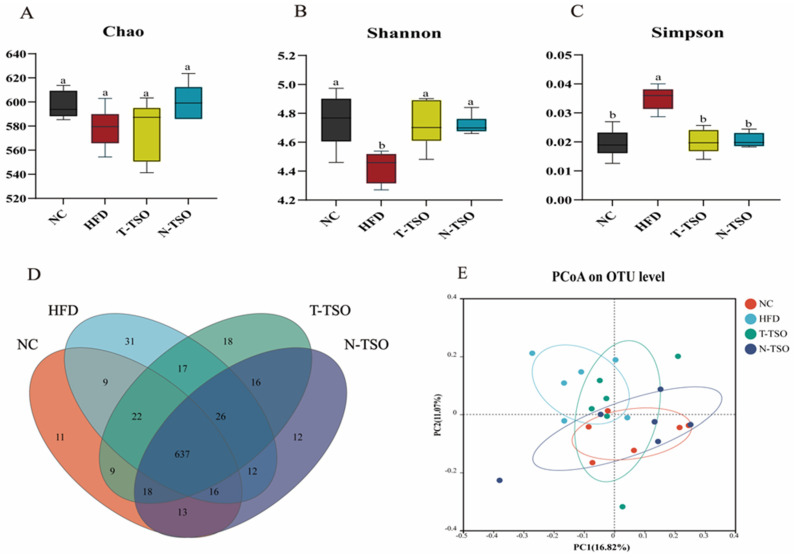
Effect of different refined tea seed oils on the diversity and structure of intestinal microbiota in obese mice induced by high-fat diet. (**A**) Chao index, (**B**) Shannon index, (**C**) Simpson index, (**D**) Venn diagram, (**E**) PCoA analysis diagram. Data are presented as the mean ± SD (n = 6). Different letters represent a significant difference among multiple groups (*p* < 0.05).

Chronic HFD intake disrupts the structural composition of intestinal microorganisms. Thus, the experiment employed 16S rRNA high-throughput sequencing to examine the impact of various refined oils on the intestinal microbiota of HFD-fed mice. In this study, alpha diversity was assessed using Chao, Shannon, and Simpson indices. The Chao index estimates the total number of microbial composition in the gut microbiota, while the Shannon and Simpson indices evaluate their diversity [40]. In Figure 4A, the Chao index showed a reduction in the HFD group compared to the NC group, indicating that HFD reduced the abundance of gut microbiota. Both T-TSO and N-TSO had the ability to increase the abundance of microbiota to some extent, restoring the HFD-damaged intestinal microbiota. Increased Shannon indices and decreased Simpson indices reflect greater microbial composition diversity. Compared with mice in the HFD group, Shannon indices significantly (*p* < 0.05) increased, and Simpson indices decreased in the NC, T-TSO, and N-TSO groups (Figure 4B,C). These findings indicated that TSO increased the abundance and microbial composition diversity of intestinal microbiota in mice, with no significant difference between T-TSO and N-TSO. 

Microbial composition differences in OTU gut bacteria from different groups were compared using Venn diagrams (Figure 4D). The total number of OTU in the NC, HFD, T-TSO, and N-TSO groups was 735, 770, 763, and 750, respectively. An overlap of 637 OTUs was found among these four groups. The number of unique OTU in the HFD group was 31, higher than the remaining three groups (11 in the NC group, 18 in the T-TSO group, and 12 in the N-TSO group). Principal coordinate analysis (PCoA) based on Bray–Curtis distance (Figure 4E) indicated that the two primary components of the gut microbial community accounted for 11.07% and 16.82% in the NC, HFD, T-TSO, and N-TSO groups, respectively. The results demonstrated a distinct separation of the HFD group from the NC group, indicating substantial (*p* < 0.05) differences in the gut microbiota. Conversely, the N-TSO group displayed a high degree of overlap with the NC group, suggesting that N-TSO intervention restored the intestinal microbiota disorder induced by HFD.

### 3.8. Microbial Composition Analyses at the Phylum Level 

**Figure 5:**
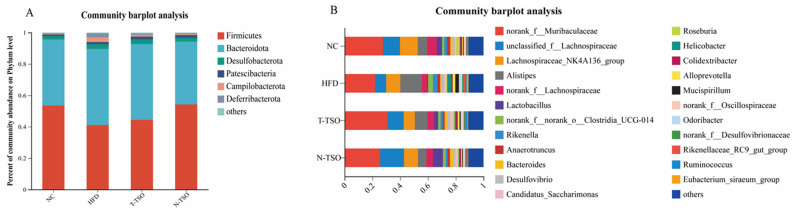
Effects of different refined tea seed oils on the intestinal microbiota of high-fat-diet-induced obese mice. (**A**) Percent community abundance diagram on phylum level and (**B**) percent community abundance diagram on genus level. Data are presented as the mean ± SD (n = 6).

There is growing body of evidence indicating that gut microbiota plays a pivotal role in the development of obesity [41]. Extra virgin olive oil (EVOO), a popular functional food and a major source of fat in the Mediterranean diet, possesses a wide range of healthy components. EVOO has been found to modulate gut microbiota composition and secondary metabolite production, ameliorating intestinal mucosal damage and obesity [42]. Recently, many plant extracts have been shown to attenuate lipid metabolism disorders and obesity syndromes by modulating the abundance and composition of the gut microbiota [43]. To further investigate the impact of TSO intervention on the intestinal microbiota of mice, we examined alterations in the intestinal microbiota at both the phylum and genus levels. At the phylum level, the predominant gut microbes include *Firmicutes*, *Bacteroidota*, *Desulfobacterota*, *Patescibacteria*, *Campilobacterota*, and *Deferribacterota*, with *Firmicutes* and *Bacteroidota* accounting for approximately 90% (Figure 5A). The ratio of *Firmicutes*/*Bacteroidetes* (F/B) is often used to reflect the obesity status, although there remains some debate in the literature. Studies involving mice and human have reported higher F/B values in obese groups compared to normal groups, and F/B values tend to decrease with weight loss following dietary intervention [6]. However, some researchers have suggested that F/B values are not necessarily linked to obesity, and, in some cases, the F/B value of obese individuals is lower than that of the normal group [44]. In our study, both the NC and tea seed oil groups exhibited reduced *Bacteroidetes* and increased *Firmicutes* when compared to the HFD group. In the N-TSO group, the abundance of *Bacteroidetes* decreased from 48.57% to 40.05%, while the *Firmicutes* increased from 41.13% to 54.24%. The F/B value was significantly (*p* < 0.05) lower in the HFD group (0.85) compared to the NC group (1.27), and it increased to 0.92 and 1.35 in the T-TSO and N-TSO groups, respectively. Evidently, the HFD had a substantial impact on the composition of gut microbiota, leading to microbial dysbiosis.

### 3.9. Microbial Composition Analyses at the Genus Level

At the genus level, HFD treatment significantly reduced the abundance of several important genera, including *norank_f__Muribaculaceae*, *unclassified_f__Lachnospiraceae*, *Lachnospiraceae_NK4A136_group*, *norank_f__Lachnospiraceae*, *Lactobacillus*, and *Bacteroides*. These genera have previously been associated with the amelioration of inflammation, the inhibition of harmful bacteria, and the promotion of anti-cancer immunity [45,46]. Lachnospiraceae NK4A136 group, in particular, is a butyrate-producing bacterium that has been shown to maintain intestinal barrier integrity in mice [47]. Lactobacillus reduces fat accumulation and can be employed to prevent obesity and intestinal ecological disorders [48]. Additionally, Bacteroides aid in the catabolism of branched-chain amino acid (BCAA) in brown adipose tissue (BAT) and play a role in preventing obesity [49]. Dietary supplementation with both T-TSO and N-TSO during HFD resulted in an increase in the abundance of these microbiota. Notably, N-TSO demonstrated a more substantial increase in the abundance of norank_f_Muribaculaceae (from 21.97% to 25.63%), Lactobacillus (from 0.73% to 7.15%), and Bacteroides (from 1.21% to 2.31%) compared to the T-TSO group (Figure 5B). In contrast, the abundance of *Alistipes*, *norank__f__norank__o___Clostridia_UCG-014*, *Rikenella*, *Helicobacter*, and *Mucispirillum* increased significantly (*p* < 0.05) after HFD intake. *Alistipes* have been found to be enriched in obese mice, promoting colorectal cell proliferation, and impairing intestinal barrier function [50]. Furthermore, *Mucispirillum* abundance was increased significantly (*p* < 0.05) in response to HFD intervention and was positively correlated with the presence of non-alcoholic steatohepatitis [51]. Nevertheless, the abundance of *Alistipes* and *Mucispirillum* could be substantially reduced after intervention with T-TSO and N-TSO. Compared to the HFD group, *Alistipes* abundance was reduced by 40.91% and 60.72%, and *Mucispirillum* abundance was reduced by 68.28% and 80.02% in the T-TSO group and N-TSO group, respectively.

### 3.10. Correlation between Obesity-Related Indicators and Gut Microbiota

**Figure 6:**
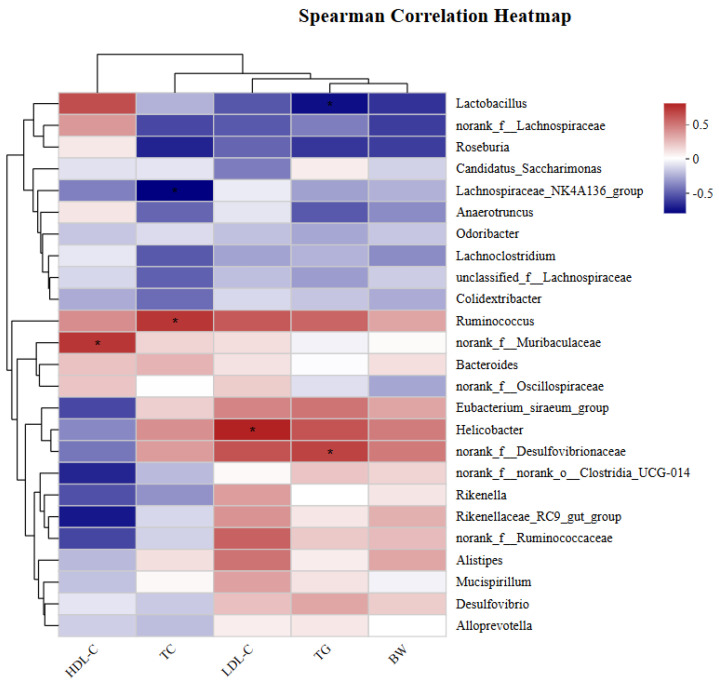
Spearman correlation analysis between the top 25 microbial genera in the intestinal flora and related parameters of mice. All data were expressed as mean ± SD (n = 6). * 0.01 ˂ *p* ≤ 0.05.

Correlations between obesity-related indices and gut microbiota were investigated with Spearman correlation analysis. As indicated in Figure 6, *Lactobacillus*, *norank_f__Lachnospiraceae*, *Roseburia*, *Lachnospiraceae_NK4A136_group*, and *Lachnoclostridium* were negatively correlated with TC, TG, LDL-C levels, and body weight. On the other hand, *Lactobacillus* and *norank_f__Lachnospiraceae* were positively correlated with HDL-C levels. These results suggest that an increase in the relative abundance of bacteria such as *Lactobacillus* and *Lachnospiraceae_NK4A136_group* can ameliorate lipid metabolism disorders and alleviate weight gain. In contrast, *Ruminococcus*, *Eubacterium_siraeum_group*, *Helicobacter*, *norank_f_Desulfovibrionaceae*, and *Alistipes* were positively correlated with TC, TG, and LDL-C levels and body weight. Therefore, the increased abundance of *Ruminococcus*, *norank_f__Desulfovibrionaceae*, and *Alistipes* may be associated with the induction of obesity and hyperlipidemia. In conclusion, our data indicate that tea seed oil can alleviate HFD-induced obesity in mice by modulating the intestinal flora and leading to a healthy intestinal flora composition similar to that in the normal group.

## 4. Conclusions

Tea seed oil contains a variety of biologically active compounds such as polyphenols, tocopherols, sterols, and squalene, which have high nutritional value. Unfortunately, many of these compounds are lost during conventional high-temperature refining processes. Therefore, we optimized the refining temperature of tea seed oil and compared the physicochemical properties of different refined tea seed oils, as well as their effects on obese mice. The data showed that tea seed oil obtained under the conditions of degumming at 70 °C, deacidification at 50 °C, decolorization at 90 °C, and deodorization at 180 °C (0.06 MPa, 1 h) effectively removed impurities while minimizing the loss of active ingredients. The loss of total polyphenols was reduced from 79.48% to 18.70%, total tocopherols from 75.24% to 15.12%, sterols from 74.79% to 10.41%, squalene from 73.14% to 16.01%, and β-carotene from 82.26% to 20.97%. In addition, the optimized tea seed oil demonstrated greater improvements in body weight and fat accumulation, lipid metabolism disorders, and liver tissue damage in obese mice compared to the traditional refined oil. Furthermore, tea seed oil was shown to modulate gut microbiota disorders induced by a high-fat diet, with notably greater normalization of microbiota abundance following N-TSO intervention. Specifically, N-TSO restored bacterial abundance and diversity, increasing some beneficial microorganisms (*norank_f__Muribaculaceae*, *Lactobacillus*, and *Bacteroides*), and decreasing some pathogenic bacteria (*Alistipes* and *Mucispirillum*). 

However, the complex interactions between tea seed oil and the gut microbiota require more evidence to reveal the exact mechanisms behind these interactions, to better understand the relationship between tea seed oil, the gut microbiota, and host health. In addition, although this study raises the possibility of using tea seed oil as an ameliorator of obesity, human clinical trials are needed to confirm the safety and efficacy of tea seed oil in ameliorating obesity. Overall, the optimized refined tea seed oil significantly reduced the loss of active ingredients and can be recommended as a dietary supplement for obese and hyperlipidemic patients.

## Figures and Tables

**Table 1:** Physicochemical properties of different-refined tea seed oil.

Parameter	Crude	N-TSO	T-TSO	CNS-TSO
Refractive index (n40)	1.478 ± 0.124	1.470 ± 0.137	1.468 ± 0.116	1.462–1.472
Acid value (mg KOH/g)	4.1 ± 0.04	0.21 ± 0.02	0.14 ± 0.06	<0.8
Iodine value (g/100 g)	87.5 ± 1.2	88.1 ± 1.4	89.2 ± 1.1	74–95
Peroxide value (mmol kg^−1^)	8.97 ± 0.64	0.85 ± 0.10	0.57 ± 0.08	<6.00
Moisture and volatile matter (%)	0.22 ± 0.07	0.07 ± 0.01	0.05 ± 0.01	<0.10
Insoluble impurities (%)	0.18 ± 0.02	0.04 ± 0.01	0.02 ± 0.01	<0.05
Phospholipids (%)	0.275 ± 0.02	0.017 ± 0.002	0.007 ± 0.001	<0.02
Fatty acid composition				
SFA (%)				
C14:0	0.18 ± 0.03	0.17 ± 0.02	0.18 ± 0.01	<0.5
C16:0	15.18 ± 0.03	15.06 ± 0.01	15.11 ± 0.01	13.0–18.0
C18:0	2.71 ± 0.01	2.63 ± 0.01	2.67 ± 0.02	2.0–6.0
C20:0	0.089 ± 0.001	0.085 ± 0.001	0.082 ± 0.001	NR
C22:0	0.044 ± 0.001	0.041 ± 0.001	0.042 ± 0.001	NR
C24:0	0.051 ± 0.001	0.048 ± 0.001	0.049 ± 0.002	NR
Total	18.25 ± 0.073	18.03 ± 0.043	18.13 ± 0.044	NR
MUFA (%)				
C16:1	0.38 ± 0.01	0.36 ± 0.01	0.37 ± 0.02	NR
C18:1	57.96 ± 0.21	57.16 ± 0.18	57.06 ± 0.23	50.0–68.0
C20:1	0.70 ± 0.002	0.68 ± 0.001	0.69 ± 0.002	NR
C22:1	0.26 ± 0.004	0.25 ± 0.003	0.25 ± 0.004	NR
C24:1	0.067 ± 0.003	0.063 ± 0.001	0.065 ± 0.002	NR
Total	59.37 ± 0.23	58.51 ± 0.20	59.44 ± 0.26	NR
PUFA (%)				
C18:2	21.13 ± 0.007	21.01 ± 0.002	21.06 ± 0.004	15.0–35.0
C18:3	0.59 ± 0.039	0.58 ± 0.028	0.57 ± 0.031	0.2–2.0
C20:2	0.014 ± 0.001	0.013 ± 0.002	0.013 ± 0.001	<1.5
Total	21.73 ± 0.047	21.60 ± 0.032	21.64 ± 0.036	NR

T-TSO: traditional refined tea seed oil; N-TSO: optimized refined tea seed oil; SFA: saturated fatty acid; MUFA: monounsaturated fatty acid; PUFA: polyunsaturated fatty acid; CNS-TSO: Chinese National Standards for tea seed oil GB/T35026-2018 [22]; NR: not required. Each value is a mean ± SD of triplicate determinations.

**Table 2:** Bioactive compounds of different refined tea seed oils.

Parameter	Crude	N-TSO	T-TSO	CNS-TSO
Total Polyphenols (mg/100 g)	36.57 ± 0.34 ^a^	29.73 ± 0.23 ^b^	7.50 ± 0.43 ^c^	NR
Total tocopherol (mg/100 g)	41.68 ± 0.35 ^a^	35.38 ± 0.45 ^b^	10.32 ± 0.24 ^c^	NR
Sterols (mg/100 g)	177.21 ± 4.38 ^a^	158.77 ± 4.84 ^b^	44.68 ± 3.45 ^c^	NR
Squalene (mg/100 g)	23.86 ± 0.18 ^a^	20.04 ± 0.58 ^b^	6.41 ± 0.48 ^c^	NR
Carotenoids (mg/100 g)	21.69 ± 0.22 ^a^	15.19 ± 0.44 ^b^	3.85 ± 0.43 ^c^	NR

T-TSO: traditional refined tea seed oil; N-TSO: optimized refined tea seed oil; CNS-TSO: Chinese National Standards for tea seed oil GB/T35026-2018; NR: not required. Each value is a mean ± SD of triplicate determinations. Means in the column followed by different superscripts (a, b, and c) are significantly different (*p* < 0.05).

## Data Availability

The original contributions presented in the study are included in the article/Supplementary Material, further inquiries can be directed to the corresponding authors.

## Supplementary Materials

The following supporting information can be downloaded at: https://www.mdpi.com/article/10.3390/foods13152352/s1, Figure S1: HPLC detection of fatty acid in N-TSO; Figure S2: HPLC detection of tocopherols in N-TSO; Table S1: HPLC detection of fatty acid in N-TSO; Table S2: Effect of temperature on deacidification; Table S3: Effect of temperature on decolorization; Table S4: Effect of temperature on deodorization; Table S5: Effect of pressure on deodorization; Table S6: Effect of time on deodorization; Table S7: Gene primer sequences.
