# Supplementary material for: Effects of Tea Seed Oil Extracted by Different Refining Temperatures on the Intestinal Microbiota of High-Fat-Diet-Induced Obese Mice
Source: Foods. 2024 Jul 26;13(15):2352. doi: 10.3390/foods13152352 (PMC11312122; doi:10.3390/foods13152352)

Effects of Tea Seed Oil Extracted by different refining temperatures on the Intestinal Flora of High fat diet-induced Obese Mice

S1. Oil refining procedures

S1.1 Methods

Degumming: Fifteen 1 kg samples of crude oil were collected and divided into 5 groups with 3 replicates each. They were heated to 25, 40, 55, 70, and 85 °C, respectively. In each sample, 100 mL of hot water at the same temperature was added for hydration degumming. The mixture was stirred slowly at 60 rpm for 15 minutes, left to stand for 3 hours, and then filtered. The degumming rate (%) was calculated as (phospholipid content of crude oil - phospholipid content of degummed oil) / phospholipid content of crude oil. The best-degummed oil was selected for the next process test.

Deacidification: Fifteen samples of degummed oil, each weighing 1 kg, were divided into 5 groups with 3 replicates. Sodium hydroxide solution (1.35 g/L) was added to the samples, shaken well after the addition of alkali, heated to 30 °C, and left to stand for 25 min. Then, 150 mL of hot water at temperatures of 20, 35, 50, 65, and 80 °C, respectively, was added to the samples, washed with slow stirring, and centrifugation was performed to remove the precipitates. The deacidification rate (%) was

calculated as (acid value of degummed oil - acid value of deacidified oil) / acid value of degummed oil. The best-deacidified oil was used for the next process test.

Decolorization: Fifteen samples of deacidified oil, each weighing 1 kg, were taken and divided into 5 groups with 3 replicates. First, the decolorized oil was heated to 40 °C, and 20 g of activated white clay was added. The temperature was then increased to 75, 90, 105, 120, and 135 °C, respectively. After gentle stirring for 80 min, the temperature was lowered to 50 °C, filtered while hot, and the absorbance was measured at 520 nm. The decolorization rate (%) was calculated as (Absorbance of deacidified oil - Absorbance of decolorized oil) / Absorbance of deacidified oil. The best-decolorized oil was used in the next process test.

Deodorization: Fifteen samples of decolorized oil, each weighing 1 kg, were stored in stainless steel containers, and divided into 5 groups. These groups were placed in a vertical pressure steam sterilizer converted deodorizer. The first heating was done to 80 °C, then evacuated to 500 Pa, and the temperature was increased to 140, 180, 220, 260, and 300 °C, respectively. After 1.5 hours, the cooling and depressurization were carried out to ambient pressure, followed by filtration to obtain the refined oil. The optimal temperature-deodorized oil was used for different deodorization time and different pressure effect tests. Fifteen decolorized oil samples of 1 kg each were taken and divided into five groups. Deodorization tests were

carried out at 0.04, 0.06, 0.1, 0.14, and 0.18 MPa, respectively. The refined oil was obtained by filtration. The optimum process-deodorized oil was used for the next process test.

S1.2 Statistical analysis

The main physicochemical parameters were statistically analyzed using Duncan's statistics, and different letters in the same column indicate significant differences ($P<0.05$).

S1.3 Results

S1.3.1 Degumming process

In the hydration degumming process, the addition of hot water at the appropriate temperature facilitates the separation of water-soluble and a smaller proportion of water-insoluble phospholipids from crude oil. Statistical analyses were conducted to assess the impact of processing at five different temperatures, revealing no significant differences in the contents of beneficial bioactive compounds (Table S1). Importantly, the phospholipid content of the degummed oils met the acceptable values specified by CNS-TSO standards. Following these findings, 70 °C was identified as the optimal degumming temperature for the process. This temperature selection ensures efficient separation of phospholipids while maintaining the integrity of beneficial bioactive compounds in the resulting oils.

Table S1. Effect of temperature on degumming

Samples	Degummed ratio (%)	Phospholipids (%)	Total Polyphenols (mg/Kg)	Total tocopherol (mg/Kg)	Total sterols (mg/Kg)	Squalene (mg/Kg)	β-carotene (mg/Kg)
Crude oil	—	0.398±0.01	365.7±3.6 [a]	416.8±4.5[a]	1772.1±43.8[a]	238.6±4.8[a]	216.9±2.2[a]
25 °C	34.6±0.80[a]		362.5±4.2[a]	412.9±6.7[a]	1767.9±23.2[a]	236.1±5.5[a]	212.6±4.0[a]
40 °C	42.33±1.58[b]		358.3±3.7[a]	416.6±4.7[a]	1760.6±22.5[a]	235.7±4.7[a]	208.3±5.3[a]
55 °C	77.33±1.91[c]		354.5±5.6[a]	410.5±5.1[a]	1754.6±16.1[a]	234.2±3.6[a]	204.7±4.2[a]
70 °C	92.93±0.78[d]		345.9±3.1[b]	399.9±3.6[a]	1749.9±29.1[a]	232.5±4.3[a]	199.3±1.8[a]
85 °C	94.2±0.98[d]		336.7±2.2[b]	390.6±4.9[a]	1554.3±27.4[b]	210.7±4.2[b]	195.8±4.1[a]

Each value is a mean ± SD of triplicate determinations. Means in the column followed by different superscripts (a, b, c, and d) are significantly different ($p < 0.05$).

S1.3.2 Deacidification process

Undesirable compounds present in degumming oil can potentially facilitate oxidation reactions, leading to undesired colors and odors in the final product. Acid removal serves to eliminate acids and other undesirable components from degumming oils. Experimental findings indicated that deacidification at 50 °C yielded the highest rate of acid removal with minimal loss of active substances. This suggests that the deacidification process at 50 °C is an effective method for achieving optimal results in terms of acid removal while preserving the integrity of essential components (Table S2).

Table S2. Effect of temperature on deacidification

Samples	Acid removal rate (%)	Acid value (mg KOH/g)	Total Polyphenols （mg/Kg)	Total tocopherol （mg/Kg)	Total sterols （mg/Kg)	Squalene （mg/Kg)	β-carotene （mg/Kg)
Degumming oil	—	2.9±0.2	345.6±6.3^{a}	400.0±3.1^{a}	1753.2±35.3^{a}	232.5±5.6^{a}	199.3±7.5^{a}
20 ℃	41.9±1.1^{a}		348.6±7.8^{a}	396.1±7.4^{a}	1740.2±36.8 ^{a}	229.3±7.6^{a}	196.2±6.6^{a}
35 ℃	76.4±3.2^{b}		339.2±7.2^{a}	392.4±2.8^{a}	1729.6±35.7^{a}	227.4±7.8^{a}	192.6±7.7^{a}
50 ℃	93.4±1.1^{c}		339.8±8.3^{a}	387.2±7.1^{a}	1715.8±35.5^{a}	225.4±8.6^{a}	187.1±5.0^{a}
65 ℃	95.0±0.6^{cd}		248.6±2.7^{b}	353.9±2.8^{b}	1447.6±44.0^{a}	189.3±2.3^{b}	151.4±3.9^{b}
80 ℃	97.1±0.6^{de}		230.2±6.1^{b}	315.6±4.1^{b}	1373.1±20.8^{b}	172.1±8.2^{b}	140.6±8.7^{b}

Each value is a mean ± SD of triplicate determinations. Means in the column followed by different superscripts (a, b, c, d, and e) are significantly different ($p < 0.05$).

S1.3.3 Decolorization process

The analysis of oils decolorized at various temperatures is presented in Table S3. The results revealed that bleaching at 75 °C was insufficient for pigment removal (bleached ratio of 53.6%). However, elevating the temperature beyond 90 ℃ (90, 105, 120, and 135 ℃) led to a significant increase in the pigment removal rate (93.3%, 94.7%, 95.8%, and 96.9% bleaching rate, respectively). Interestingly, the average bleaching rate of these decolorized oils showed no significant change as the temperature increased from 90 °C to 120 °C. Nevertheless, it is noteworthy that the content of bioactive compounds, such as total polyphenols, total tocopherols, sterols, and squalene, experienced a drastic decrease above 90 °C. Consequently, 90 °C bleaching was determined as the optimal process, striking a balance between effective pigment removal and the preservation of essential bioactive compounds.

Table S3. Effect of temperature on decolorization

Samples	Bleached ratio (%)	Total Polyphenols（mg/Kg)	Total tocopherol（mg/Kg)	Total sterols（mg/Kg)	Squalene（mg/Kg)	β-carotene（mg/Kg)
Deacidified oil	—	339.8±5.3^{a}	386.7±2.1^{a}	1715.8±17.8^{a}	225.4±5.1^{a}	187.1±3.4^{a}
75 ℃	53.6±1.9^{a}	332.8±3.6^{a}	382.4±3.3^{a}	1720.7±43.5 ^{a}	221.5±4.1^{ab}	178.3±4.8^{b}
90 ℃	93.3±2.2^{b}	332.3±6.2^{a}	369.3±5.8^{b}	1645.9±33.7^{a}	216.7±1.9^{b}	169.3±3.0^{c}
105 ℃	94.7±0.9^{bc}	311.4±6.2^{b}	305.3±5.0^{c}	1583.3±27.0^{a}	194.9±5.3^{c}	151.2±2.6^{d}
120 ℃	95.8±0.5^{bc}	268.2±9.9^{c}	281.8±3.5^{d}	1304.3±56.6^{b}	173.7±5.9^{d}	140.7±5.4^{e}
135 ℃	96.9±1.2^{c}	246.6±8.3^{d}	314.6±3.8^{c}	1240.8±31.2^{c}	151.6±1.8^{e}	120.3±5.8^{f}

Each value is a mean ± SD of triplicate determinations. Means in the column followed by different superscripts (a, b, c, d, e, and f) are significantly different ($p < 0.05$).

S1.3.4 Deodorization process

In the deodorization process, the removal of volatile compounds responsible for unpleasant tastes and odors in decolorized oils is achieved through high temperature and high pressure. The oil decolored at 90 °C underwent deodorization at 0.1 MPa for 60 minutes at five different temperatures (140, 180, 220, 260, and 300 °C). Results demonstrated that deodorization at 140 °C was insufficient for effectively removing volatile compounds. However, a significant increase in the removal of volatile compounds was observed by raising the temperature above 180 °C (Table S4). Despite the enhanced removal of volatile compounds, it was noted that specific flavors and active substances in the oil gradually decreased with increasing temperature. Consequently, 180 °C was selected as the optimal deodorization temperature, balancing the removal of undesired compounds while preserving essential qualities.

Further exploration of the optimal deodorization pressure and time

involved selecting different pressures (0.04, 0.06, 0.08, 0.10, and 0.12 MPa) and durations (30, 60, 120, 180, and 240 minutes) for treating the decolored oil at 180 °C. Results indicated that the decolorized oil significantly removed undesirable volatile substances while retaining some of the tea seed oil flavor when treated at 180 °C and 0.06 MPa (Table S5) for 1 h (Table S6). Importantly, this condition resulted in the lowest loss of total polyphenols, total tocopherols, sterols, squalene, and β-carotene in the oil.

60 min, 0.1 MPa

Table S4. Effect of temperature on deodorization.

Samples	Flavors	Odours	Total Polyphenols (mg/Kg)	Total tocopherol (mg/Kg)	Total sterols (mg/Kg)	Squalene (mg/Kg)	β-carotene (mg/Kg)
Decolourised oil	Bitter taste	smelly	$332.3±6.2^{a}$	$376.0±9.8^{a}$	$1635.9±20.6^{a}$	$216.7±8.1^{a}$	$169.3±5.2^{a}$
140 °C	Bitter taste	smelly	$328.2±5.9^{a}$	$371.9±5.3^{ab}$	$1588.2±8.2^{ab}$	$212.7±3.6^{a}$	$164.0±3.3^{ab}$
180 °C	No bitter taste	Slightly characteristic odour of TSO	$298.7±5.4^{b}$	$365.1±9.3^{ab}$	$1608.1±28.5^{b}$	$209.1±8.7^{a}$	$159.4±9.8^{ab}$
220 °C	No bitter taste	Odourless	$242.9±5.3^{c}$	$359.5±6.8^{b}$	$1541.3±11.1^{c}$	$203.5±6.1^{a}$	$154.2±1.3^{b}$
260 °C	No bitter taste	Odourless	$196.1±5.7^{d}$	$229.7±3.4^{c}$	$989.1±13.4^{d}$	$170.3±3.8^{b}$	$86.6±6.5^{c}$
300 °C	No bitter taste	Odourless	$185.4±2.9^{e}$	$204.7±5.1^{d}$	$870.1±10.4^{e}$	$135.0±9.4^{c}$	$53.8±4.9^{d}$

Each value is a mean ± SD of triplicate determinations. Means in the column followed by different superscripts (a, b, c, d, and e) are significantly different ($p < 0.05$).

180 °C, 60 min

Table S5. Effect of pressure on deodorization.

Samples	Flavors	Odours	Total Polyphenols (mg/Kg)	Total tocopherol (mg/Kg)	Total sterols (mg/Kg)	Squalene (mg/Kg)	β-carotene (mg/Kg)
Decolourised oil	Bitter taste	smelly	$335.6±3.1^{a}$	$376.0±2.8^{a}$	$1645.9±33.7^{a}$	$216.7±1.9^{a}$	$169.3±3.0^{a}$
0.04 MPa	Bitter taste	smelly	$330.1±2.4^{a}$	$373.4±3.3^{a}$	$1634.4±30.8^{a}$	$214.1±3.2^{a}$	$168.7±5.0^{a}$
0.06 MPa	No bitter taste	Slightly characteristic odour of TSO	$326.6±4.9^{ab}$	$373.0±5.3^{a}$	$1622.1±25.8^{a}$	$210.5±4.1^{a}$	$165.2±3.4^{a}$

0.10 MPa	No bitter taste	Odourless	318.0±4.2^{b}	360.1±2.4^{b}	1521.7±19.1^{b}	210.3±2.2^{b}	152.2±2.0^{b}
0.14 MPa	No bitter taste	Odourless	269.2±1.9^{c}	340.4±1.2^{c}	1499.4±38.1^{b}	199.7±4.1^{b}	140.6±5.3^{c}
0.18 MPa	No bitter taste	Odourless	228.4±2.9^{d}	283.6±4.8^{d}	1394.8±28.2^{c}	175.8±3.5^{c}	130.4±2.1^{d}

Each value is a mean ± SD of triplicate determinations. Means in the column followed by different superscripts (a, b, c, and d) are significantly different ($p < 0.05$).

180 ℃, 0.06 MPa

Table S6. Effect of time on deodorization.

Samples	Flavors	Odours	Total Polyphenols （mg/Kg)	Total tocopherol （mg/Kg)	Total sterols （mg/Kg)	Squalene （mg/Kg)	β-carotene （mg/Kg)
Decolorized oil	Bitter taste	smelly	332.3±6.2^{a}	376.0±5.8^{a}	1645.9±33.7^{a}	216.7±1.9^{a}	169.3±3.0^{a}
30 min	Bitter taste	smelly	330.5±4.6^{a}	374.3±4.4^{a}	1641.7±40.1^{a}	215.6±4.5^{a}	168.9±2.4^{a}
60 min	No bitter taste	Slightly characteristic odour of TSO	328.6±2.3^{a}	370.5±9.4^{a}	1637.6±36.7^{a}	213.9±3.3^{a}	163.0±3.5^{a}
120 min	No bitter taste	Odourless	301.1±3.2^{b}	351.4±7.5^{b}	1497.5±39.6^{b}	200.4±1.2^{b}	150.1±4.2^{b}
180 min	No bitter taste	Odourless	260.8±3.1^{c}	274.1±5.3^{c}	1369.8±35.3^{c}	181.2±4.1^{c}	133.9±3.2^{c}
240 min	No bitter taste	Odourless	230.4±4.9^{d}	248.2±2.6^{d}	1229.8±23.0^{d}	165.3±1.7^{d}	113.9±5.4^{d}

Each value is a mean ± SD of triplicate determinations. Means in the column followed by different superscripts (a, b, c, d, e, and f) are significantly different ($p < 0.05$)

S1.4 Conclusion

Tea seed oil is rich in nutritional content, including vital bioactive compounds such as polyphenols, tocopherols, sterols, squalene, and beta-carotene. Unfortunately, conventional high-temperature refining processes often lead to the loss of these valuable compounds. In our research, we've discovered that reducing the operating temperature during various refining steps is an effective approach to remove unwanted impurities and preserve bioactive compounds, thereby maintaining the nutritional integrity of tea seed oil, all within the same processing time and vacuum pressure.

Based on the outcomes of our studies, we have opted for a

combination process in our experimental study. This process involves degumming at 70 °C, deacidification at 50 °C, decolorization at 90 °C, and deodorization at 180 °C (0.06 MPa, 1 hour). This selected combination aims to strike a balance between effective purification, minimal loss of bioactive compounds, and the preservation of the nutritional value of tea seed oil.

S2. qRT-PCR analysis

Table S7 Gene primer sequences

Gene		Primer sequence (5' to 3')
PPAR-α	Forward	GTTCGCAGCTGTTTTGGGG
	Reverse	GAACTTCAACTTGGCTCTCCT
FAS	Forward	GCGGGTTCGTGAAACTGATAA
	Reverse	GCAAAATGGGCCTCCTTGATA
SREBP-1c	Forward	CTTTGGCCTCGCTTTTCGG
	Reverse	TGGGTCCAATTAGAGCCATCTC
β-action	Forward	AGTGTGACGTTGACATCCGT
	Reverse	GCAGCTCAGTAACAGTCCGC

Figures

Figure S1. HPLC detection of fatty acid in N-TSO

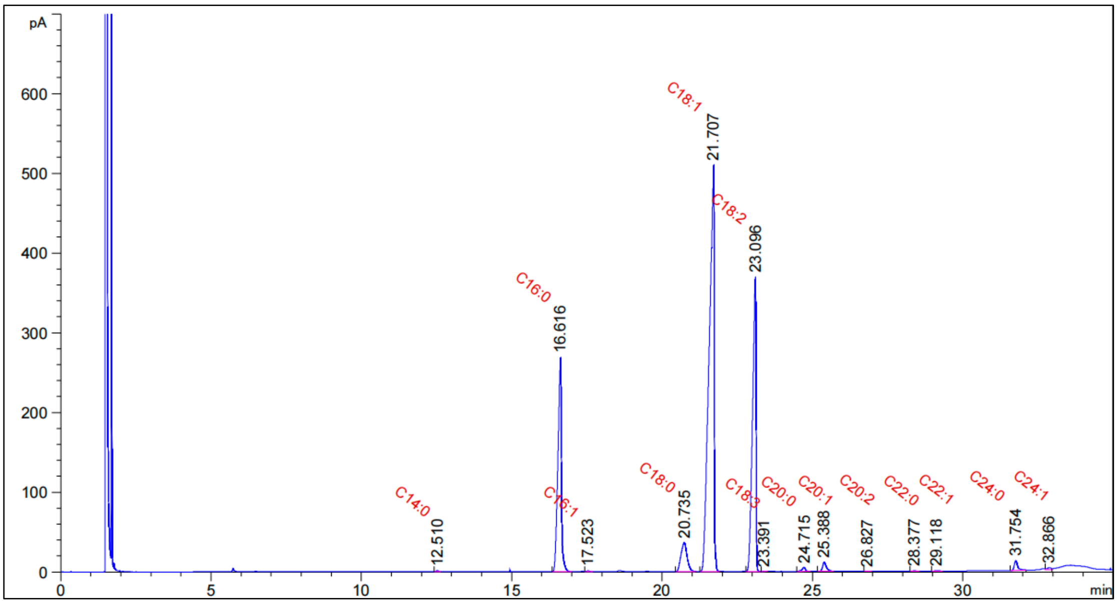

Figure S2. HPLC detection of tocopherols in N-TSO

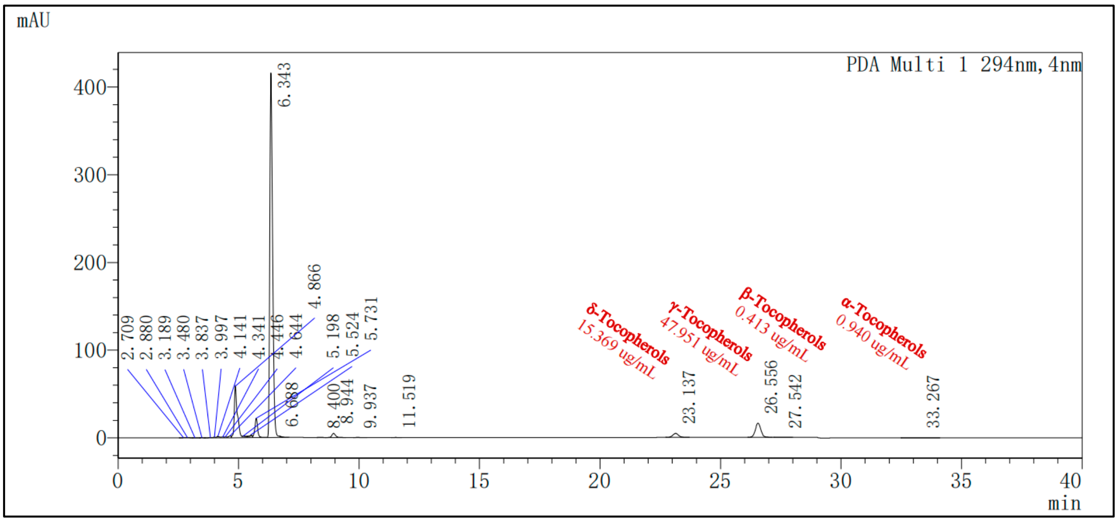

Supplement: Supplementary file 1 [file foods-13-02352-s001.zip › foods-3081090-supplementary.pdf]
